# Supplementary figures and images for: Promoting Photosynthetic Production of Dammarenediol-II in Chlamydomonas reinhardtii via Gene Loading and Culture Optimization
Source: Int J Mol Sci. 2023 Jul 2;24(13):11002. doi: 10.3390/ijms241311002 (PMC10341690; doi:10.3390/ijms241311002)

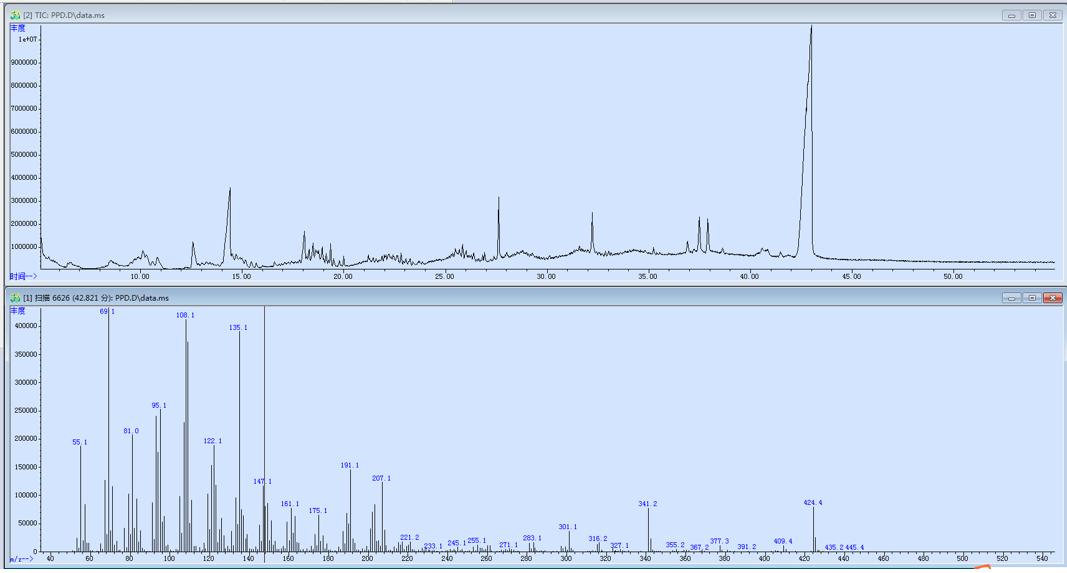

Supplement: Supplementary file 1 [file ijms-24-11002-s001.zip › Supplementary Figure S1.tif]

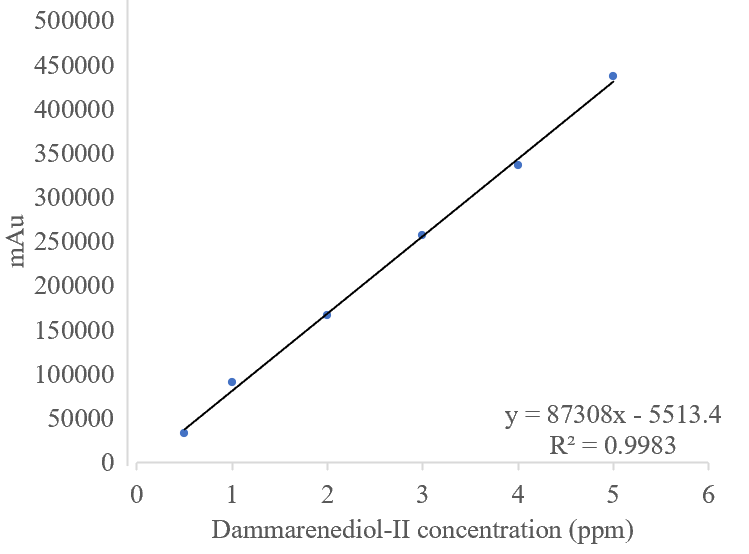

Supplement: Supplementary file 1 [file ijms-24-11002-s001.zip › Supplementary Figure S2.tif]
